# Lactation Affects Isolated Mitochondria and Its Fatty Acid Composition but Has No Effect on Tissue Protein Oxidation, Lipid Peroxidation or DNA-Damage in Laboratory Mice

**DOI:** 10.3390/antiox5010002

**Published:** 2016-01-11

**Authors:** Teresa G. Valencak, Johannes Raith, Katrin Staniek, Lars Gille, Alois Strasser

**Affiliations:** 1 Research Institute of Wildlife Ecology, University of Veterinary Medicine, Savoyenstrasse 1, Vienna A-1160, Austria; 2 Institute of Physiology, Pathophysiology and Biophysics, University of Veterinary Medicine, Veterinärplatz 1, Vienna A-1210, Austria; alois.strasser@vetmeduni.ac.at; 3 Institute of Veterinary Public Health, University of Veterinary Medicine, Veterinärplatz 1, Vienna A-1210, Austria; Johannes.raith@vetmeduni.ac.at; 4 Institute of Pharmacology and Toxicology, University of Veterinary Medicine, Veterinärplatz 1, Vienna A-1210, Austria; katrin.staniek@vetmeduni.ac.at (K.S.); lars.gille@vetmeduni.ac.at (L.G.)

**Keywords:** lactation, protein oxidation, TBARS, isolated mitochondria, oxygen consumption, comet assay, mitochondrial fatty acid composition, C57BL/6NCrl

## Abstract

Linking peak energy metabolism to lifespan and aging remains a major question especially when focusing on lactation in females. We studied, if and how lactation affects *in vitro* mitochondrial oxygen consumption and mitochondrial fatty acid composition. In addition, we assessed DNA damage, lipid peroxidation and protein carbonyls to extrapolate on oxidative stress in mothers. As model system we used C57BL/6NCrl mice and exposed lactating females to two ambient temperatures (15 °C and 22 °C) while they nursed their offspring until weaning. We found that state II and state IV respiration rates of liver mitochondria were significantly higher in the lactating animals than in non-lactating mice. Fatty acid composition of isolated liver and heart mitochondria differed between lactating and non-lactating mice with higher *n*-6, and lower *n*-3 polyunsaturated fatty acids in the lactating females. Surprisingly, lactation did not affect protein carbonyls, lipid peroxidation and DNA damage, nor did moderate cold exposure of 15 °C. We conclude that lactation increases rates of mitochondrial uncoupling and alters mitochondrial fatty acid composition thus supporting the “uncoupling to survive” hypothesis. Regarding oxidative stress, we found no impact of lactation and lower ambient temperature and contribute to growing evidence that there is no linear relationship between oxidative damage and lactation.

## 1. Introduction

Linking peak energy metabolism to lifespan and aging remains a major task that has intrigued scientists for a long time. Conceivably, mitochondria as a cell’s single greatest source of energy but also as net producer of reactive oxidative species (ROS) are of major interest in this context. Within mitochondria, the main source of ROS during oxidative phosphorylation is the electron transport chain. The amount of ROS produced depends on the state of respiration in the mitochondria and also on the activity of uncoupling proteins (UCPs) [1]. UCPs uncouple oxidative phosphorylation and generate heat instead of ATP. This process is generally referred to as non-shivering thermogenesis that is mediated by UCPs and characterized by uncoupled respiration. Non-shivering thermogenesis is the primary source of metabolic heat and takes place mainly in brown adipose tissue found in all small placental mammals and representing a very old phylogenetic trait. When uncoupling occurs, the respiratory chain is accelerated and less ROS are produced per unit O_2_ consumed, a concept that was summarized under “uncoupling to survive” [2]. Undoubtedly then, a decrease in ROS production should affect metabolism at most during peak phases of energy expenditure such as lactation in mammals but surprisingly few studies are available on this topic. In this contribution we thus aimed to test if mitochondrial metabolism and respiration would be altered during lactation by affecting oxidative stress in the females. Additionally, we intended to test if exposition to a lower ambient temperature during this phase would boost mitochondrial metabolism. Raising offspring represents the most energy-demanding process for female mammals [3] during which females produce a lot of heat through the exothermic process of milk production and increased food intake (reviewed in [4]). In parallel, non-shivering thermogenesis is largely shut down [5] to limit hyperthermia during lactation [6]. Do these fundamental changes in metabolism affect mitochondrial respiration, and, if so, to what extent? At this end, we also were interested in effects of these changes on known side effects of chronic and intense production of ROS on macromolecules such as DNA, proteins and lipids. We thus hypothesized that by manipulating mitochondrial respiration during reproduction we might impact on the female’s “rate of living” and also assessed different parameters related to oxidative stress such as DNA damage, protein and lipid oxidation. Firstly, we harvested and isolated mitochondria from liver and heart tissues to measure mitochondrial respiration from both reproducing and control females. To specifically address the role of a lower ambient temperature on mitochondrial respiration, we exposed two of our four experimental groups to a lower ambient temperature of 15 °C. By doing so, we aimed to test the “rate of living hypothesis” along with the identifying differences in mitochondrial respiration. We tested if fatty acid composition would change in isolated mitochondria either related to lactation or not or according to differing ambient temperatures of 22 °C or 15 °C. To elucidate the role of potentially altered mitochondrial respiration on long-term macromolecular damage we assessed DNA damage (by applying the comet assay) on spleen lymphocytes, measured protein oxidation by detecting protein carbonyls in brain tissue and kidneys and finally quantified lipid peroxidation (by quantifying thiobarbituric acid reactive substances (TBARS)) on kidney and lung tissue as well as on spleen lymphocytes. The 3-weeks long process of lactation in laboratory mice most likely imposes a chronic oxidative stress burden on the animals’ biological macromolecules, which we aimed to quantify indirectly in view of the often very unspecific reactions in tissues that hinder identification of single ROS molecules.

Undoubtedly, and as recently highlighted by Pichaud *et al.* (2013) [7], the capacity of mitochondria to generate ATP might determine to a large extent whether or not a lactation event is successful in a female. In our study, presented here we therefore aimed to shed more light on changes in mitochondrial respiration imposed by lactation through observation of preparatory changes of fatty acid composition and potential effects on oxidative stress.

## 2. Experimental Section

### 2.1. Animals and Housing

A total of twenty-two, 2–4 months old female C57BL/6NCrl mice from Charles River Laboratories, Bad Sulzfeld, Germany were used for the experiment. Females from the delivered batch of mice that did not become pregnant after pairing were used as non- lactating mice. F1-female offspring from the litters were paired in the next round. Mice were assigned to four experimental groups. Two groups consisted of females raising offspring at 22 ± 2 °C and 15 ± 2 °C while two groups served as non-lactating mice under the same thermal conditions. Please note that all the females from the F1 generation used in our study were from litters raised at 22 °C. All animals were housed individually in polycarbonate cages (365 × 207 × 140 mm, Eurostandard type II Long, Tecniplast, Buguggiate, Italy) equipped with sawdust (Lignocel FS14, JRS, Rosenberg, Germany), some nesting material and red mouse houses (Tecniplast, Italy) as enrichment material. All females had ad libitum access to rodent breeding chow (V118x, Sniff, Soest, Germany) and water throughout the experiment. The metabolisable energy content of this diet amounted to 14.3 MJ/kg at 23% raw protein, 6% raw fat, 3.3% raw fiber, 6.8% raw ashes, 49.2% nitrogen free extracts, 34% starch and 5.2% sugar. All mice were kept on a 16 h/8 h light/dark photoperiod with lights on at 6 am. Their thermal environment was thus either a climate chamber that was running at 15 ± 2 °C or, normal room temperature conditions of 22 ± 2 °C. Before, during pairing and for most of pregnancy the females of all four experimental groups were kept at room temperature conditions of 22 ± 2 °C. Only on day 14, three days before the first expected parturitions, females exposed to 15 °C were moved to the climate chamber that is located just next to the “warm” animal room, has the same 8-fold air exchange per hour just as the light regime but has an efficient cooling and alarming system to ensure the temperature is kept constant throughout the day and is monitored permanently. When females gave birth, the exact number of pups was noted down and animals were left undisturbed for the duration of lactation that we terminated on day 18 when the offspring were weaned, weighed and separated from their mothers. The average litter size in reproducing females was between 5 and 7 per female and was not manipulated in our experiment. No significant differences were found in both mean litter size and/or mass among the groups. On the day of weaning, we sacrificed the females (see below) and sampled liver, heart, kidneys, lungs, brain and spleen lymphocytes for later analyses (see below). Non-lactating mice were sacrificed in the same manner and sample collection was done accordingly. Right after the dissections, samples (except livers, hearts and spleen lymphocytes) were flash frozen in liquid nitrogen and stored at −80 °C until consecutive biochemical analyses (see below).

### 2.2. Isolation of Liver and Heart Mitochondria

Mice were killed by cervical dislocation and immediately after, the livers and hearts were removed and placed into a beaker with chilled buffer. This buffer contained 250 mM sucrose, 5 mM Tris and 2 mM ethylene glycol tetraacetic acid (EGTA) with pH set at 7.4 at 4 °C. Tissues were manually homogenized with scissors and then transferred over into a glass tissue homogenizer (Potter Elvehjem) and were homogenized in buffer with a rotating teflon pestle. The homogenate was poured into a 50 mL centrifuge tube, filled up with buffer and prepared for centrifugation. We used a refrigerated centrifuge (Sorvall RC 26 plus equipped with SS 34 rotor, Thermofisher, Waltham, MA, USA) for the following three centrifugation steps (the first one with 2500 rpm and the second and third one with 9000 rpm) of 10 min each while operating at 4 °C. The centrifugation and the intervening procedure were standardized for the purpose of isolating the liver and heart mitochondria from the tissue homogenates. After isolation of mitochondria was completed, protein content in each sample was determined using a standard protocol for Biuret determination using bovine serum albumin (BSA) as standard.

### 2.3. Measurement of Mitochondrial Respiration Rates

To measure oxygen consumption rates, we used a Clark-type oxygen electrode (Oxygraph system, Hansatech Instruments, Norfolk, UK). The reaction vessel of the electrode was constantly surrounded by a water bath kept at 37 °C to mimic body temperature of individual mice. The first step after calibration was adding buffer into the reaction vessel. This buffer contained 120 mM KCl, 3 mM HEPES, 1 mM EGTA, 5 mM KH_2_PO_4_ and 0.3% defatted BSA with pH set at 7.4 at 37 °C. Afterwards mitochondria were added at a concentration of 0.5 mg protein/mL. Then we carefully closed the reaction vessel with the standard plug and made sure that no air bubbles remained in the vessel. Once the vessel was closed this was considered the beginning of *State I* respiration. In the next step we added 10 µL glutamate-malate as substrates to a final concentration of 5 mM each using a Hamilton syringe and measured *State II respiration*. *State III* was induced with the addition of 5 µL 500 mM ADP (adenosine diphosphate) and after complete conversion of ADP to ATP (adenosine triphosphate) *State IV* respiration was assessed. Immediately after the measurements we extracted the raw data using the Oxygraph system (Hansatech Instruments, Norfolk, UK). Respiratory control ratios (RCRs) were determined as the ratio of oxygen consumption during *State III* and *State IV* respiration. The calculated values were subsequently classified after group of animals, entered into a spreadsheet and analyzed statistically.

### 2.4. Fatty Acid Composition of Isolated Mitochondria

Isolated mitochondria homogenates of liver and heart samples were stored in Eppendorf cups at −80 °C until analysis (<2 weeks). Lipids were extracted from the liver and heart isolated mitochondria homogenates (250 μL each) by using chloroform and methanol (2:1, *v*/*v*). They were separated on silica gel thin layer chromatography plates (Kieselgel 60, F254, 0.5 mm, Merck Millipore, Darmstadt, Germany) and then made visible under ultraviolet light with the phospholipid fraction isolated and removed. In both heart and liver mitochondria homogenates, we measured the composition of total phospholipids that obviously combines all subcellular membranes in one measurement as previously noted down in Valencak and Ruf (2013) [8]. The phospholipid extracts were transesterified by heating (100 °C) for 30 mins and extracted into hexane. Fatty acid methyl esters were identified by gas-liquid chromatography using a Perkin Elmer Auto System XL with FID and Auto Sampler (Norwalk, CT, USA). We used a 30 m × 25 mm HP INNOWax capillary column (Hewlett Packard, CA, USA). The temperature parameters were set to elute all fatty acid methyl esters that were identified by comparing retention times with those of FA methyl ester standards (Sigma-Aldrich, St. Louis, MO, USA). Peaks were integrated using the Turbochrom Software (Perkin Elmer, Norwalk, CT, USA).

### 2.5. Measurements of DNA Damage

For assessing DNA damage in our four experimental groups we used the comet assay for cytotoxicity testing from spleen lymphocytes of the females (with little modifications as previously described in more detail by Strasser *et al.* (2012) [9]). An appropriate cell suspension (40 µL) was diluted in 400 µL 0.5% low-melting point agarose and 100 µL were pipetted on glass slides pre-coated with 1% high-melting point agarose. After lysis (performed in pre-chilled lysis solution) slides were placed in Comet assay tank^®^ (Trevigen Inc., Gaithersburg, MA, USA) and incubated in pre-chilled electrophoresis buffer. Electrophoresis was performed at 25 V, 300 mA for 20 min in the comet assay tank (chilled with ice). Afterwards slides were washed in neutralization buffer and purified H_2_O, fixed in 80% ethanol and stained following the Comet assay silver staining kit^®^ (Trevigen Inc.) instructions. As suggested by Wiklund and Agurell (2003) [10] two slides were prepared for each sample. Fifty randomly chosen cells were scored manually per slide (total 100 cells). Comet assay parameters such as “olive moment” (summation of tail intensity profile values multiplied by their relative distances to the head center, divided by total comet intensity), “tail moment” (% DNA in tail multiplied by tail length) and % DNA in tail (total tail intensity divided by total comet intensity; multiplied by 100) were analyzed by CometScore^®^ (TriTek Corp, Summerduck, VA, USA).

### 2.6. Measurement of Protein Oxidation

Protein oxidation was quantitated spectrophotometrically from kidneys, brains, lungs and spleen lymphocytes of the females as described in detail by Fagan *et al.* (1999) [11]. Specifically, carbonyl groups were quantitated by using 2,4-dinitrophenylhydrazine (DNPH). In brief, tissue samples were homogenized, filtered and fractionated to separate soluble and insoluble proteins and protein content of fractions was determined. As buffer we used pyrophosphate relaxing buffer (PRB) as described in Fagan *et al.* (1999) [11]. Heme contaminants were removed by washing with trichloroacetic acid (TCA) and ethanol-ethylacetate. 500 μL 10 mM DNPH (dissolved in 2 M·HCl) were added to 500 μL of the sample containing a known amount of tissue protein in PRB for reaction with the protein carbonyl groups (at room temperature in dark) [11]. Sample blanks were prepared using defined amounts of sample in PRB and 2 M·HCl with no DNPH. Both the samples and the sample blanks were run in duplicates. After addition of the DNPH solution, the tubes were vortexed every 10 min for a period of 1 h. TCA was then added to each tube. The tubes were vortexed, placed in an ice-bath and then spun in a microcentrifuge. After centrifugation, the supernatant was decanted and 1 mL of ethanol-ethylacetate (1:1, *v*/*v*) solution was added to each tube. Following mechanical disruption of the pellet by vortexing, the tubes were allowed to stand for 10 min and then spun again to remove unreacted DNPH. The supernatant was decanted and the pellet washed with ethanol-ethylacetate. After the final wash, the protein was solubilized in 1 mL 6 M guanidine hydrochloride and 20 mM potassium dihydrogen phosphate (pH = 2.3). The final solution was centrifuged to remove any insoluble material. The carbonyl content was calculated from the absorbance measurement at 380 nm and an absorption coefficient ε = 22,000 M^−1^·cm^−1^.

### 2.7. Measurement of Lipid Peroxidation

Finally, we measured lipid peroxidation by determining the level of thiobarbituric acid reactive substances (TBARS) from kidney, brain and lung tissue as well as from spleen lymphocytes according to the method described by Vincent *et al.* (2005) [12], with modifications. In brief, tissue samples were weighed out ~20 mg, sonicated in 200 µL radioimmunoprecipitation assay buffer supplemented with 50 µL protease inhibitor cocktail (Sigma P8340 aprotinin, AEBSF, bestatin, E-64, leupeptin, pepstatin A) per 50 mL assay buffer and 0.1% digitonin and centrifuged at 3000 g for 10 min and 4 °C. Then 10 μL aliquots for protein analysis were removed and thereafter 100 μl sample was added to 200 μL ice-cold 10% trichloroacetic acid on ice for 15 min to precipitate protein. Precipitated samples were centrifuged at 2200 g for 15 min at 4 °C. Supernatants were mixed with an equal volume of 0.67% thiobarbituric acid and then boiled for 15 min. Once cooled the absorbance was read at 532 nm on an absorbance plate reader (PerkinElmer EnSpire 2300 multilabel Reader). 1,1,3,3-tetramethoxypropane in the range of 0–100 μmol/L was used as standard.

### 2.8. Statistical Analysis

Statistical analyses were done in R 3.1.1. All data and their residuals were normally distributed so parametric testing was done. In the first analyses we computed general linear models and 2-way interactions including factors such as cold exposure (15 °C) *vs.* room temperature (22 °C), lactation *vs.* control for all measured parameters. However as we pooled the ambient temperature data, we did not include interactions except for tissue x lactation. Further, in all models individual body weight as well as individual age ranging from 2 to 7 months was included. When necessary we log-transformed the parameters (states of respiration for liver and heart mitochondria). We found no effect of ambient temperature (15 °C *vs.* 22 °C) in any of the measured parameters. Thus, for all provided tables and figures, we pooled the data into two groups and focused on the comparison between lactating (*n* = 12) and non-lactating mice (*n* = 10). For protein carbonyls and TBARS we run more than one assay per individual and therefore data were not independent from each other. We computed linear mixed effects models (package “lme4” [13]) to account for this. Statistical significance was set at *p* ≤ 0.05 in all cases. In tables and figures, mean values ± standard errors of the mean (SEM) are given.

### 2.9. Ethics

All experiments described here were approved by the ethics committee of the University of veterinary medicine and the Austrian Ministry of Science prior to the study on 24th March 2008 (GZ 68.205/0065-II/10b/2008) and comply with the laws in Austria.

## 3. Results

### 3.1. Mitochondrial Respiration

**Figure 1:**
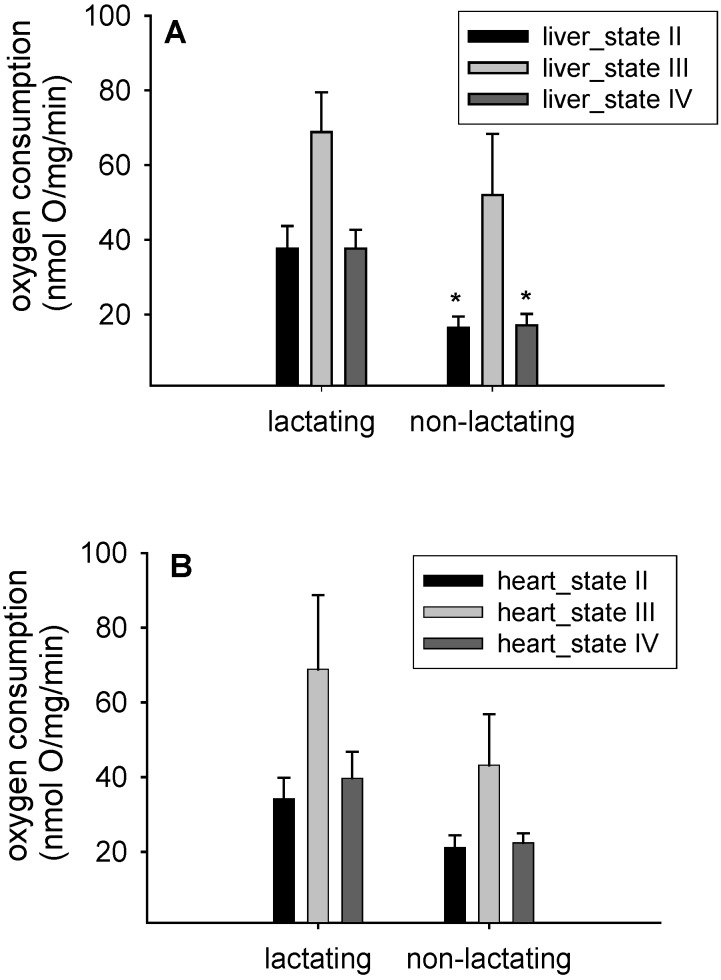
Oxygen consumption rates of isolated liver mitochondria (**A**) and isolated heart mitochondria (**B**) from lactating (*n* = 12) and control mice (*n* = 10). Data are means ± SEM; * *p* < 0.05 compared to lactating mice.

The biggest observed differences on different states of respiration between the four experimental groups were observed for the States II and IV in the liver mitochondria of lactating *versus* non-lactating individuals (state II: F_1,16_ = 10.7, *p* = 0.004; state IV: F_1,16_ = 12.7; *p* = 0.003, Figure 1A). This corresponded to mean values of 37.7 ± 20.9 nmol·O/mg/min for lactating and 16.4 ± 9.5 nmol·O/mg/min in non-lactating mice for state II (Figure 1A). Please note that ambient temperature did not affect the respiration rates (state II: F_1,16_ = 0.5; *p* = 0.49; data not shown). Age of the females did not affect mitochondrial respiration (e.g., liver state IV respiration: F_1,16_ = 0.005; *p* = 0.9; data not shown).

In contrast, data from heart mitochondria failed to reach significance for any of the respiratory states (e.g., state II: F_1,15_ = 1.8; *p* = 0.2; Figure 1B). Yet, the mean values for heart mitochondrial state II respiration were pointing to the same direction as in liver with 34.2 ± 19.5 nmol·O/mg/min in lactating mice and 21.1 ± 10.5 nmol·O/mg/min in non-lactating mice. Again, we detected no difference between different ambient temperatures (F_1,15_ = 0.4; *p* = 0.5; data not shown). Liver and heart mitochondrial respiration control ratio (RCR) and protein concentrations are shown in Table 1. While we observed no difference in heart RCR’s between lactating and non-lactating females, liver RCR’s were higher in the non-lactating mice compared to lactating mice (Table 1).

**Table 1:** Respiratory control ratios (State III/State IV) and protein concentrations of isolated liver and heart mitochondria from lactating and non-lactating C57BL/6NCrl mice. Data are means ± SEM.

		Lactating Mice	Non-Lactating Mice
*Respiratory control ratio (nmol·O/mg/min)*	Liver mitochondria	1.87 ± 1.2	3.75 ± 0.98
	Heart mitochondria	1.9 ± 0.78	1.96 ± 1.3
*Protein concentration (mg/mL)*	Liver mitochondria	77.6 ± 9.64	75.4 ± 5.53
	Heart mitochondria	20.2 ± 1.9	26.8 ± 2.4

### 3.2. Fatty Acid Composition of Isolated Liver and Heart Mitochondria

**Figure 2:**
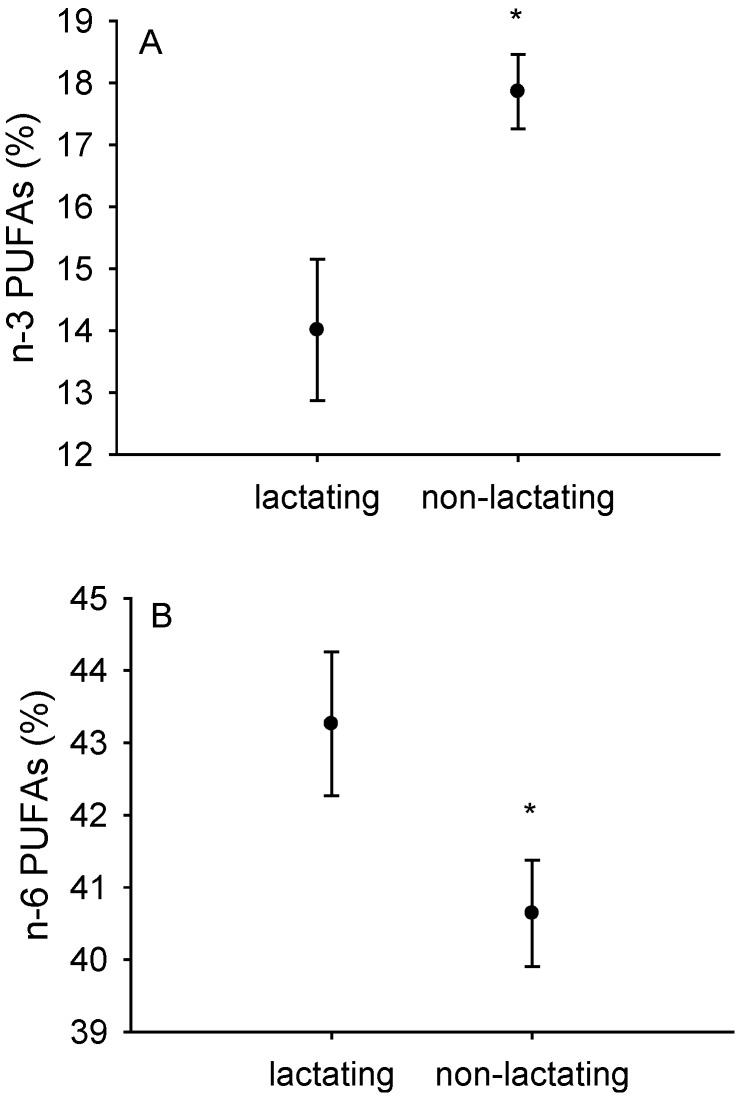
Content of *n*-3 (**A**) and *n*-6 (**B**) polyunsaturated fatty acids in isolated liver mitochondria from lactating (*n* = 12) and non-lactating (*n* = 10) mice. Data are means ± SEM; * *p* < 0.05, in comparison to lactating mice (F_1,6_ = 66.04; *p* = 0.0002).

In liver mitochondria we observed that reproductive state affected *n*-3 polyunsaturated fatty acid content (partial for reproductive state: F_1,6_ = 66.04; *p* = 0.0002, Figure 2A). Ambient temperature had no influence (F_1,6_ = 0.0006; *p* = 0.9; data not shown). As can be seen from Figure 2, *n*-6 polyunsaturated fatty acids increased in lactating females (partial for reproductive state: F_1,6_ = 15.4; *p* = 0.008, Figure 2B) while the *n*-3 decreased. This effect was mirrored in isolated heart mitochondria (*n*-3 fatty acids partial for reproductive state: F_1,8_ = 45.7; *p* = 0.0001; Table 2). In isolated liver mitochondria we observed mean values of *n*-3 polyunsaturated fatty acids of 14.1% ± 1.1% in lactating and 17.9% ± 0.6% in non-lactating mice (Table 2) whereas we observed even higher means of 17.5% ± 2.3% in isolated heart mitochondria from lactating females (Table 2). Interestingly, the amount of the very long-chained and highly unsaturated fatty acid C 22:6*n*-3 amounted to 12.5% in liver mitochondria of lactating and 16.8% in non-lactating mice (Table 2).

**Table 2:** Fatty acid composition of phospholipids in liver and heart mitochondria of lactating (L) and non-lactating control females (C). Values are given in weight %, means ± SEM.

Liver Mitochondria			Heart Mitochondria	
	L	C	L	C
N	12	10	12	10
**C 14:0**	0.06 ± 0.003	0.04 ± 0.0003	0.3 ± 0.02	0.3 ± 0.003
**C 15:0**	0.03 ± 0.002	0.04 ± 0.002	0.05 ± 0.003	0.05 ± 0.003
**C 16:0**	20.9 ± 0.64	19.8 ± 0.4	18.2 ± 0.4	19.3 ± 1.01
**C 17:0**	0.3 ± 0.01	0.3 ± 0.02	0.3 ± 0.003	0.3 ± 0.01
**C 18:0**	15.4 ± 0.84	16.7 ± 0.7	24.3 ± 0.09	25.1 ± 0.3
**C 16:1n-7**	0.4 ± 0.03	0.3 ± 0.03	0.6 ± 0.03	0.5 ± 0.05
**C 18:1n-9**	5.7 ± 0.32	4.4 ± 0.5	8.9 ± 0.3	5.9 ± 0.4
**C 18:2n-6**	17.2 ± 0.03	16.3 ± 0.3	19.4 ± 1.3	11.6 ± 0.3
**C 18:3n-3**	0.09 ± 0.01	0.08 ± 0.01	0.13 ± 0.01	0.12 ± 0.09
**C 20:4n-6**	26.1 ± 0.04	24.4 ± 0.5	10.3 ± 0.4	6.1 ± 0.24
**C 20:5n-3**	0.69 ± 0.04	0.49 ± 0.04	0.48 ± 0.06	0.24 ± 0.0003
**C 22:5n-3**	0.7 ± 0.03	0.5 ± 0.02	1.5 ± 0.06	1.1 ± 0.22
**C 22:6n-3**	12.5 ± 0.4	16.8 ± 0.2	15.9 ± 0.6	29.7 ± 1.6

### 3.3. DNA Damage

We observed olive tail moment was similar between lactating and non-lactating females (partial for reproductive state: F_1,15_ = 2.2; *p* = 0.16; Table 3). Equally, there was no effect of ambient temperature on DNA damage in lymphocytes (F_1,15_ = 0.9; *p* = 0.36; data not shown).

**Table 3:** DNA damage in lactating and non-lactating C57BL/6NCrl mice. Data are means ± SEM.

	Lactating Mice	Non-Lactating Mice
Tail DNA (%)	78.9 ± 2.6	68.93 ± 2.7
Tail Moment (%)	133.5 ± 9.3	128.4 ± 7.8
Olive Tail Moment (%)	64.3 ± 4.3	59.3 ± 3.04

#### Protein Oxidation

The amount of protein oxidation quantitated via protein carbonyls did not differ between reproducing and non-lactating females (partial for reproductive state: F_1,15_ = 0.007; *p* = 0.9; Table 4). There was no significant partial effect of cold exposure (F_1,15_ = 0.2; *p* = 0.64). Protein carbonyls slightly differed between tissues with spleen lymphocytes showing the highest values in lactating females (partial for tissue: F_2,42_ = 3.3; *p* = 0.049; Table 4).

**Table 4:** Protein carbonyls and TBARS (means ± SEM) from lactating and non-lactating C57BL/6NCrl mice.

	Brain	Kidney	Lung	Spleen Lymphocytes
**Lactating L**				
*Protein carbonyls* (nmol/mg)	2.71 ± 0.3	1.99 ± 0.4	1.96 ± 0.3	3.75 ± 0.4
*TBARS* (nmol/mg)	45.81 ± 5.2	15.498 ± 2.6	52.795 ± 5.7	47.635 ± 7.7
**Non reproducing controls C**				
*Protein carbonyls* (nmol/mg)	3.12 ± 0.5	2.39 ± 0.5	1.71 ± 0.3	2.49 ± 0.1
*TBARS* (nmol/mg)	53.98 ± 14.4	23.17 ± 3.9	50.54 ± 4.6	36.63 ± 5.3

### 3.4. Lipid Peroxidation (TBARS)

We compared lipid peroxidation between three tissues, kidney, lung, brain and spleen lymphocytes and observed it was highest in brain and lung (partial for respectively used tissue: F_2,40_ = 21.15; *p* = 0.0001, Table 4). Yet, there were no differences between lactating and non-lactating mice (F_1,16_ = 0.8; *p* = 0.38) and no influence of ambient temperature (F_1,16_ = 1.45; *p* = 0.246). Finally, no interaction was found between reproductive state and tissue (F_1,16_ = 1.6; *p* = 0.21).

## 4. Discussion

### 4.1. Mitochondrial Metabolism and Fatty Acid Composition in Lactation

Lactation clearly causes significant energetic costs to females [4,14,15]. Conceivably, changes in energy allocation and expenditure affect cellular structure and function as well. We show that *in vitro* mitochondrial oxygen consumption was higher in lactating females than in non-lactating mice (Figure 1). While our findings were much more dominant in liver mitochondria than in heart, we are confident that the same trend existed in heart mitochondria but failed to reach significance due to differences in mitochondrial protein concentration and a higher variability between individuals that might relate to lactation itself but also possibly might arise from behavioral differences between individually kept non-lactating mice and somewhat group-housed lactating females with their litters. We speculate that due to lactation, organ sizes of liver and hearts might have been enlarged due to phenotypic plasticity [16] and these organ size enlargements or hypertrophy may have even further complicated our *in vitro* measurements of mitochondrial respiration. Another confirmation for altered mitochondrial metabolism in both hearts and livers of lactating mice was that fatty acid composition differed in both tissues (Figure 2, Table 2). Lactating females had lower *n*-3 polyunsaturated fatty acids and more *n*-6 polyunsaturated fatty acids. Clearly, these changes might have given rise to altered fatty acid utilization during lactation [17]. Our working hypothesis that lactating C57BL/6NCrl mice have higher rates of uncoupling than non-lactating mice was therefore confirmed. Equally, the concept of mitochondrial hormesis or “mitohormesis” suggested by Schulz *et al.* (2007) seems relevant in view of our findings [18]. It is well known that *n*-3 polyunsaturated fatty acids such as eicosapentaenoic (C 20:5*n*-3) and docosahexaenoic acid (C 22:6*n*-3) are essential nutrients for mammals and can only be obtained from the diet. In our study, experimental mice were fed the same diet, which clearly contained a certain proportion of *n*-3 polyunsaturated fatty acids (as can be seen from mitochondrial fatty acid composition data, Table 2). Whether or not, and, to what extent, however selective mobilization of *n*-3 fatty acids from the pregnant female to the foetuses might have affected our observed mitochondrial fatty acid composition remains unanswered but clearly deserves further attention in the future. Possibly, n-3 polyunsaturates are diverted preferentially to the foetuses to improve brain development or neurogenesis in general while the female is pregnant. In addition, they might be diverted to the mammary gland for milk production during lactation. This may, at least in part, lead to a relatively decreased pool of essential *n*-3 polyunsaturated fatty acids in the lactating females compared to the non-lactating mice (Figure 2A,B). This is also confirmed in Table 2. In the lactating females, the important *n*-3 docosahexaenoic acid (C 22:6*n*-3) was replaced by arachidonic acid (C 20:4*n*-6), and by linoleic acid (C 18:2*n*-6) and arachidonic (C 20:4*n*-6) in liver and heart mitochondrial membrane phospholipids respectively.

Surprisingly, we did not identify any additive effect of cold exposure on oxygen consumption of isolated mitochondria. We speculate that there was no capacity left to further modulate mitochondrial oxygen consumption once the levels of lactation are reached. Corresponding to this, Pichaud *et al.* (2013) [7] observed that larger litter sizes at peak lactation did not affect mitochondrial respiration. We thus interpret that the degree to which females invest into lactation does not impact on mitochondrial respiration any further. Yet, we propose that the adjustments occurring in isolated mitochondria from lactating females might further contribute to the processes occurring around peak lactation, *i.e.*, sustained energy intake [4,5,6]. Therefore, far, the approximate 2 °C increase in body temperature in lactating females [6] was mostly attributed to processes such as milk synthesis and peak foraging and energy absorption rates. With our new data obtained in this study, we are proposing that mitochondrial respiration states further contribute to heat-stress and the risk of potential over-heating during lactation, commonly referred to as the “heat dissipation limit” (reviewed in [4]). However, to confirm this, future studies in lactating females are needed to separate the role of decreased non-shivering thermogenesis in the brown adipose tissue from rates of uncoupling in other tissues such as liver and heart. Our results point to a an increase in state II and state IV respiration so electron transport clearly was increased while ATP synthesis was secondary during these states when mitochondria were respiring on substrate (glutamate/malate in our case) yet no ADP was added (during state II) or ADP was used up (state IV). This process of “uncoupled” respiration might be affected by changes in fatty acid composition of isolated liver and heart mitochondria. Our observation that *n*-3 fatty acids are decreased in the lactating animals while the *n*-6 fatty acid class is increased (Table 2) may indicate that polyunsaturated fatty acids may play a role as signaling molecules in this process. Clearly, with respect to mitochondrial fatty acid composition, lower *n*-3 polyunsaturated fatty acids point to a lowered peroxidisability of mitochondrial membranes just as observed in membranes of long-lived Ames dwarf mice [8] and in long-lived mammals in general [19]. Membrane fatty acid composition appears to be a genetically-regulated parameter, specific for each particular animal species, although we have minimal understanding of the mechanisms responsible. This is largely supported by the observation that strains of mice differing in longevity have different membrane fatty acid composition even though maintained throughout their entire life in the same environment [20,21]. Whether the altered mitochondrial fatty acid composition in lactating females was related either to their peak metabolism or to the anti-oxidative response fighting back increased ROS production thus remains unanswered. In addition, we are suggesting that the relationship between mitochondrial membrane fatty acid composition and longevity is not simple and deserves further attention. Yet, we can confirm that lactating females have “uncoupled” mitochondria with indices such as respiration control ratio (RCR) being 50% less in liver mitochondria of lactating females (Table 1). In addition, we add that during this phase of lactation, fatty acid composition in isolated mitochondria was switched to a pattern that resembles that of membranes found in metabolically less active tissues or those of long-lived mammals [8,19]. Future studies on the impact of fatty acids during uncoupled respiration in lactation are needed to disentangle the effects of these changes on rates of lactation metabolism and long-term consequences on lifespan.

### 4.2. Oxidative Stress and Lactation

According to the resource allocation concept [22] (reviewed in Speakman and Garrat, 2014 [23]) the production of oxidative stress was blamed the proximal factor explaining some, if not overall costs of reproduction. Thus, it is widely assumed that reproduction leads to increased levels of oxidative stress, which may constrain reproduction and survival in the long run. Specifically, oxidative stress life history theory proposes that ROS are produced in direct proportion to metabolic rate resulting in oxidative stress and damage to macromolecules [24]. However, meanwhile quite a few studies have observed that oxidative stress was unchanged or even lower in reproductive individuals in comparison with those that did not reproduce [25,26] (reviewed in Speakman and Garrat, 2014 [23]). Hypothetically, oxidative stress results from an imbalance between anti-oxidative protection and production of ROS. If therefore experimental data from lactating females reveal ambiguous results, most likely there is no clear increase in oxidative stress during reproductive events in small rodents such as laboratory mice. Rather, we have to reject the hypotheses that higher energy expenditures during lactation do affect DNA-damage (Table 3), the amount of protein carbonyls (Table 4) or TBARS (Table 4). Even further and to this point, we observed that exposing lactating females to 15 °C only and thereby increasing their maternal energy demands manifold did not significantly affect the results (*p*-values all > 0.05). Albeit not measured by us in this study, we hypothesize that females rather up-regulated antioxidant protection mechanisms such as increasing catalase activity [25], superoxide dismutase [27] and glutathione [22] to compensate for the potentially higher rates of oxidative stress.

Pichaud *et al.* (2013) [7] suggested that lactation-associated changes in metabolism might lead to an increase in ROS production. Yet, animals might subsequently upregulate antioxidant defence activity measures and thus decrease oxidative stress [7]. Similarly, also others observed that antioxidant activities were regulated physiologically in response to the elevated ROS production in several tissues during peak lactation [26]. Were our results obtained and published a decade ago, the resource allocation framework with direct costs of reproduction had much more support due to the lack of experimental evidence that has been produced meanwhile. To date, we have to conclude that maternal investment into young most likely increases oxidative stress during the process of lactation although this might be restricted to certain markers and/or tissues [28]. As recently proposed by Blount *et al.* (2015) [28] a process called “oxidative shielding” may protect females or specifically their foetuses and gametes from oxidative stress well before the birth of young. While we can only refer to the process of lactation with our current study, future studies need to quantitatively assess oxidative stress during conception and pregnancy.

## 5. Conclusions

We conclude that lactation significantly increased *in vitro* mitochondrial oxygen consumption and rates of mitochondrial uncoupling. We found no higher rates of uncoupling in lactating females exposed to 15 °C. Thus, we suggest that lactating females of the C57BL/6NCrl strain already reached maximal capacity in their liver and heart mitochondria. Our new observation that the amount of *n*-6 polyunsaturated fatty acids in isolated liver and heart mitochondria was increased in lactation while *n*-3 fatty acids were found to be lower in females points to an interesting adaptation of metabolism during lactation that deserves further investigation. Interestingly, our study failed to provide support for the concept of increased oxidative stress during reproduction. Rather, we observed no changes in DNA-damage, the amount of protein carbonyls and TBARS in tissues of lactating females. We interpret our findings in view of currently accumulating evidence that metabolism in reproducing females is well prepared for the challenges of lactation and either fights back, tolerates or even decreases ROS production. Future studies will need to identify the role of polyunsaturated fatty acids in mitochondria that support the abovementioned processes.
